# Occupational Fatigue: Relationship With Personality Traits and Decent Work

**DOI:** 10.3389/fpsyg.2021.742809

**Published:** 2021-09-08

**Authors:** Annamaria Di Fabio, Andrea Svicher, Alessio Gori

**Affiliations:** ^1^Department of Education, Languages, Intercultures, Literatures and Psychology (Psychology Section), University of Florence, Florence, Italy; ^2^Department of Health Sciences, (Psychology Section), University of Florence, Florence, Italy

**Keywords:** occupational fatigue, occupational acute fatigue, occupational chronic fatigue, occupational persistent fatigue, personality traits, decent work

## Abstract

Psychology of working theory (PWT) and psychology of working framework (PWF) offered a psychological view of decent work. The present study examined the associations among personality traits, decent work and *Occupational Fatigue Exhaustion Recovery Scale* (OFER). Two hundred and thirty four participants filled out the *Big Five Questionnaire*, the Italian version of the *Decent Work Scale*, and the Italian version of the *OFER Scale*. Hierarchical regressions showed that decent work explained incremental variance beyond personality traits with respect to OFER both considering total score and its three dimensions (chronic fatigue, acute fatigue, persistent fatigue). The present study underlined the value of decent work in relation to occupational fatigue beyond the contributions of personality, in particular in relation to the dimensions of *Adequate compensation* and *Free time and rest* for less occupational fatigue (both as total and as dimensions).

## Introduction

Occupational fatigue is a global challenge. It is estimated that 35% of employees in the EU (Eurofound, 2017) and 38% in the USA (Ricci et al., 2007) are affected by fatigue associated with work. Similar issues exist in Eastern countries (Yun et al., 2008; Guo et al., 2017; Kachi et al., 2020) and developing countries (Sabir and Isha, 2016; Krishnamurthy et al., 2017; Choobineh et al., 2018). The consequences of occupational fatigue are highly relevant, affecting employees' psychological and physical health (Rose et al., 2017; Lock et al., 2018), absenteeism from work (Sagherian et al., 2019), organizational productivity (Reynolds et al., 2004; Rosekind et al., 2010), and safety concerns (Techera et al., 2016).

There is consensus among scholars that occupational fatigue is more detrimental when it becomes chronic (Roy-Byrne et al., 2002; Dansie et al., 2012; Kuehn, 2018; Zhang et al., 2019). On the basis of the literature, fatigue and stress seem reciprocally associated; the development of one can predict the insurgence of the other, with recovery acting positively in reducing both (e.g., Winwood et al., 2005, 2006; Doerr et al., 2015; Blustein et al., 2016). According to this principle, Winwood et al. (2005, 2006) provided an extensively used (e.g., Somantri et al., 2020; Rutledge et al., 2021) and comprehensive classification of occupational fatigue, distinguishing between acute fatigue, chronic fatigue, and persistent fatigue (Winwood et al., 2005, 2006).

Acute fatigue is an adaptive transient state of energy depletion resulting from workload (Winwood et al., 2005, 2006). Chronic fatigue is a long-term maladaptation to work due to a high level of stress without adequate recovery (Winwood et al., 2005, 2006). Persistent fatigue deals with the absence of optimal intershift recovery that, in turn, facilitates the insurgence of chronic fatigue (Winwood et al., 2005, 2006).

Recent studies have suggested that occupational fatigue may be explained by personality traits (Saksvik-Lehouillier et al., 2012; Vassend et al., 2018; Sørengaard et al., 2019). Personality is a stable pattern of individual characteristics that shape cognition, emotions, behavior, and motivation (Costa and McCrae, 1992) and may influence fatigue in terms of personal vulnerability (Costa et al., 2000). The majority of personality studies have focused on the five-factor model, which advances five facets of personality: extraversion, agreeableness, conscientiousness, emotional stability (or its opposite, neuroticism), and openness to experience (Costa and McCrae, 1992, 1995). Previous results have shown that neuroticism is positively associated with fatigue (De Vries and Van Heck, 2002; Calderwood and Ackerman, 2011; Vassend et al., 2018; Sørengaard et al., 2019) and chronic fatigue (Deary and Chalder, 2010; Poeschla et al., 2013; Valero et al., 2013). Furthermore, individuals with high neuroticism feel exhausted more frequently and report more severe fatigue than those with lower neuroticism (Kangas and Montgomery, 2011). Similarly, emotional stability has been found to be negatively associated with fatigue, and findings have shown that lower levels of this trait have a negative impact on fatigue (Kitamura et al., 2013). Previous results have also indicated that conscientiousness has a positive relationship with fatigue (De Vries and Van Heck, 2002; Besharat et al., 2011; Calderwood and Ackerman, 2011; Sørengaard et al., 2019) and exhaustion (Alarcon et al., 2009). However, in a recent study by Sørengaard et al. (2019), conscientiousness did not predict fatigue. Most studies have shown that extraversion is negatively related to fatigue and that workers with higher extraversion tend to feel lower fatigue and chronic fatigue than individuals with low extraversion (De Vries and Van Heck, 2002; Nater et al., 2010; Besharat et al., 2011; Poeschla et al., 2013). Concerning openness, (Nater et al., 2010) compared individuals with chronic fatigue and healthy subjects and found no differences in this latter trait. Similarly, Besharat et al. (2011) reported no association between openness and fatigue in individuals with chronic fatigue. Prior studies have shown that emotional stability and extraversion make a greater contribution to fatigue than does openness (e.g., De Vries and Van Heck, 2002; Calderwood and Ackerman, 2011; Vassend et al., 2018).

The recently developed psychology of working theory (Blustein, 2013; Duffy et al., 2016; Blustein et al., 2018, 2019; PWT), which began as the psychology of working framework (PWF; Blustein, 2006), includes a social justice perspective (Blustein et al., 2018, 2019), in relation to the contemporary work scenario (Autin et al., 2020; Blustein et al., 2020). The aim of the PWT is to enhance social inclusion by supporting decent work for every person (Guichard, 2009; Peiró and Tetrick, 2011; Di Fabio and Blustein, 2016; Blustein et al., 2018; Di Fabio and Kenny, 2019; Duffy et al., 2020). PWT researchers have presented a psychological view of decent work based on the existing standards developed by the International Labor Organization (International Labour Organization, 2015). In this framework, decent work comprises characteristics related to the psychological perspective of employees' quality of work-life (Duffy et al., 2017). Decent work describes a set of five job characteristics: (1) physically and psychologically safe working conditions, (2) adequate compensation, (3) sufficient rest and free time, (4) organizational values that complement family and social values, and (5) reasonable access to healthcare (Duffy et al., 2016, 2017). The fulfillment of these characteristics fits the definition of decent work (Duffy et al., 2017). According to the PWT framework, employees can become more exhausted when they work long hours, lack healthcare coverage, and experience physically and psychologically insecure working conditions (Duffy et al., 2021).

Several studies have examined decent work characteristics in relation to occupational fatigue. According to the job demands-resources (JDR) model, indecent work (i.e., physically and psychologically unsafe work, as well as prolonged physical and psychological engagement) contributes to stress, thereby leading to burnout, of which fatigue is a component (Bakker and de Vries, 2021). Other results have highlighted the relationship between physically and psychologically unsafe work and occupational fatigue (Preckel et al., 2005; Bakker et al., 2008) and stress-related disorders (Nieuwenhuijsen et al., 2010; Jung et al., 2020). Likewise, several studies have shown that individuals working in jobs characterized by long working hours with a low frequency of breaks (e.g., Tucker, 2003; Williamson and Friswell, 2013) or insufficient recovery between shifts are more likely to suffer fatigue (Åkerstedt et al., 2002; Thorsteinsson and Brown, 2009; Williamson and Friswell, 2013). Other literature has identified a connection between inadequate compensation (i.e., low salary and extra shifts without compensation) and high occupational stress conditions (Glazer and Gyurak, 2008; Zbryrad, 2009; Liu and Onwuegbuzie, 2012) and burnout (Keinan and Malach-Pines, 2007). There is no evidence of a relationship between organizational values complementing family and social values and occupational fatigue. However, several results have stressed that work–family conflict could derive from work demands intruding into daily family life (Nomaguchi, 2009; Crain et al., 2014; Reichl et al., 2014; Hanif Abdul et al., 2016) could be associated with fatigue (Reichl et al., 2014). Access to healthcare is another critical element of decent work associated with occupational fatigue (Duffy et al., 2021). Employees who underutilize preventive care services seem to suffer more from chronic fatigue and medical-related pathologies (Lerman et al., 2012; Richter et al., 2016; Duffy et al., 2019, 2021).

Two studies directly examined the relationship between decent work and occupational fatigue (Duffy et al., 2017; Di Fabio and Kenny, 2019) and found that decent work dimensions were negatively correlated with occupational fatigue. Specifically, sufficient rest and free time had the highest correlation (Di Fabio and Kenny, 2019) and may play a central role in the connection between decent work and occupational fatigue (Duffy et al., 2017). Furthermore, PWT researchers have proposed that workers with decent jobs suffer less occupational fatigue and have healthier conditions (Duffy et al., 2021). Consequently, it seems critical to investigate which characteristics of decent work are related to occupational fatigue, taking into account relevant individual features, such as personality traits.

To our knowledge, no research has thus far studied occupational fatigue, personality traits, and decent work relationships. In particular, no data are available on the specific contribution of decent work beyond personality traits in relation to occupational fatigue. Thus, this research aimed to study the specific contribution of dimensions of decent work (i.e., safe working conditions, access to healthcare, adequate compensation, free time and rest, and complementary values) beyond personality traits (extraversion, agreeableness, conscientiousness, emotional stability, openness) in relation to occupational fatigue variables (total occupational fatigue, chronic fatigue, acute fatigue, and persistent fatigue).

According to the previous literature framework, we advance the following hypotheses:

**H1**. Emotional stability and extraversion are negatively associated with chronic fatigue, acute fatigue, persistent fatigue, and total occupational fatigue.
**H2**. Conscientiousness, openness, and agreeableness are not associated with chronic fatigue, acute fatigue, persistent fatigue, or total occupational fatigue.
**H3**. Decent work is negatively associated with chronic fatigue, acute fatigue, persistent fatigue, and total occupational fatigue.
**H4**. Sufficient rest and free time contribute prominently to lower levels of occupational fatigue (i.e., negative and statistically significant associations with chronic fatigue, acute fatigue, persistent fatigue, and total occupational fatigue).

## Methods

### Participants

The participants in this research were 234 Italian workers belonging to different public and private organizations of Tuscany (females = 57%, males = 43%; mean age = 45.05 years, SD = 11.75). Participants were predominantly white Italian regular workers recruited in a voluntary manner from the organizations, which gave their permission for workers to participate in this study.

### Measures

#### Occupational Fatigue Exhaustion Recovery Scale—Italian Version

The Occupational Fatigue Exhaustion Recovery (OFER) scale (Winwood et al., 2005, 2006; Italian version: Di Fabio, 2018) is a 15-item self-administered questionnaire measuring occupational fatigue over the last few months, using a Likert scale ranging from 0 (*strongly disagree*) to 6 (*strongly agree*).

The questionnaire encompasses a total score and three subscales: acute fatigue (OFER-AF) (sample item: “I usually feel exhausted when I get home from work”), chronic fatigue (OFER-CF) (sample item: “I often dread waking up to another day of my work”), and recovery/persistent fatigue (OFER-IR) (sample item: “I rarely recover my strength fully between work periods”). The OFER offers two interpretations of scores on the OFER-IR subscale: recovery (i.e., effective recovery between shifts), in which negative items are recoded, and persistent fatigue (i.e., lack of recovery between shifts), in which positive items are recoded. In this study, we consider persistent fatigue scores. The OFER subscales have been shown to have Cronbach's alpha coefficients between 0.83 and 0.89 (Winwood et al., 2006). The Italian version of the OFER (Di Fabio, 2018) has adequate psychometric properties in line with the original scale. In this research, the reliability coefficients were 0.95 for total score, 0.87 for OFER-AF, 0.83 for OFER-CF, and 0.73 for OFER-IR.

#### Big Five Questionnaire

The five-factor personality traits (extraversion, agreeableness, conscientiousness, emotional stability, openness) were detected through the Big Five Questionnaire (BFQ; Caprara et al., 1993), which comprises 132 items ranked on a Likert scale from 1 (*absolutely false*) to 5 (*absolutely true*). The questionnaire showed excellent reliability (Cronbach's alpha coefficients between 0.73 and 0.90). In the current study, the reliability coefficients in were 0.76 (extraversion), 0.80 (agreeableness), 0.84 (conscientiousness), 0.90 (emotional stability), and 0.75 (openness).

Examples of items are: “If necessary, I don't mind helping a stranger” (Agreeableness); “It is easy for me to talk to strangers” (Extraversion); “I am always informed about what is going on in the world” (Openness); “I take care of things, even the smallest details” (Conscientiousness); “I do not usually over-react, even in the presence of strong emotions”(Emotional Stability).

#### Decent Work Scale—Italian Version

The Decent Work Scale (DWS Duffy et al., 2017; Italian version: Di Fabio and Kenny, 2019) is a self-report tool designed to assess decent work. It includes 15 items ranked on a Likert scale from 1 (*strongly disagree*) to 7 (*strongly agree*) with five dimensions: safe working conditions (sample item: “I feel emotionally safe interacting with people at work”), access to healthcare (sample item: “I get good healthcare benefits from my job”), adequate compensation (sample item: “I am not properly paid for my work”), free time and rest (sample item: “I do not have enough time for non-work activities”), and complementary values (sample item: “The values of my organization match my family values”). The Italian version adapted the dimension of access to healthcare considering the Italian context (Di Fabio and Kenny, 2019). The original scale showed good reliability (Cronbach's coefficients between 0.79 and 0.97) (Duffy et al., 2017). The psychometric properties of the Italian version of the DWS are in line with the original version (Di Fabio and Kenny, 2019). In the current study, the reliability coefficients were 0.83 (safe working conditions), 0.95 (access to healthcare), 0.84 (adequate compensation), 0.74 (free time and rest), and 0.90 (complementary values).

#### Procedure

Participation was anonymous, and confidentiality was guaranteed. Data collection consisted of the OFER scale, the BFQ, and the DWS. General information regarding the purposes of the research was conveyed to the participants beforehand. The research was carried out according to Italian privacy and informed consent norms, and the procedure was in line with ethical standards approved by the Ethics Committee of the Integrated Psychodynamic Psychotherapy Institute (IPPI).

#### Data Analysis

The variables were checked for normality (asymmetry and kurtosis), and values in the range of ±1 were considered adequate (Tabachnick and Fidell, 2012). The values of the 14 variables in the present study did not exceed these thresholds. Pearson correlations were then calculated for the study variables. Descriptive statistics were reported as means with standard deviation. Subsequently, four hierarchical linear regressions were run in relation to occupational fatigue. The first three hierarchical regressions each used one of the OFER subscales (i.e., chronic fatigue [OFER-CF], acute fatigue [OFER-AF], persistent fatigue [OFER-IR]) as dependent variables. The last hierarchical regression used the OFER total score as a dependent variable. The five BFQ dimensions were entered in Step 1. Then, the five DWS dimensions were added in Step 2. To control for the influence of multicollinearity, the variance inflation factor (VIF) for every independent variable was analyzed. Values lower than 2 were considered adequate. Each study variable had a value lower than 2. Statistical analysis was performed with IBM SPSS Statistics (Version 21). Significance levels were established at *p* < 0.05 (two-tailed).

## Results

Table 1 shows the zero-order Pearson correlation of the study variables. Extraversion and emotional stability showed statistically significant and negative correlations with all OFER subscales and total score (Table 1). Conscientiousness showed statistically significant and negative correlations with OFER-CF, OFER-IR, and total score, but not with OFER-AF (Table 1). Agreeableness and openness did not show statistically significant correlations with the OFER subscales or total score (Table 1).

**Table 1:** Zero order pearson correlation.

	**1**	**2**	**3**	**4**	**5**	**6**	**7**	**8**	**9**	**10**	**11**	**12**	**13**	**14**
1 BFQ E	–													
2 BFQ A	0.10	–												
3 BFQ C	0.34^**^	0.08	–											
4 BFQ ES	0.22^**^	0.18^**^	0.17^*^	–										
5 BFQ O	0.36^**^	0.36^**^	0.30^**^	0.25^**^	–									
6 DWS SW	0.16^*^	0.11	0.14^*^	0.20^**^	0.13^*^	–								
7 DWS AH	0.05	0.24^**^	−0.02	0.05	0.08	0.34^**^	–							
8 DWS AC	−0.02	−0.03	0.05	0.09	−0.07	0.22^**^	0.19^**^	–						
9 DWS FT	−0.02	0.03	−0.04	0.11	−0.06	0.05	0.13^*^	0.23^**^	–					
10 DWS CV	0.10	0.13^*^	0.10	0.01	−0.01	0.38^**^	0.34^**^	0.23^**^	0.05	–				
11 OFER-AF	−0.22^**^	0.01	−0.10	−0.29^**^	0.00	−0.24^**^	−0.11	−0.30^**^	−0.40^**^	−0.21^**^	–			
12 OFER-CF	−0.26^**^	−0.12	−0.22^**^	−0.32^**^	−0.13	−0.29^**^	−0.22^**^	−0.26^**^	−0.24^**^	−0.19^**^	0.63^**^	–		
13 OFER-IR	−0.24^**^	−0.11	−0.18^**^	−0.29^**^	−0.05	−0.26^**^	−0.13	−0.33^**^	−0.40^**^	−0.16^*^	0.74^**^	0.56^**^	–	
14 OFER Tot	−0.28^**^	−0.09	−0.19^**^	−0.35^**^	−0.07	−0.31^**^	−0.18^**^	−0.33^**^	−0.39^**^	−0.21^**^	0.90^**^	0.86^**^	0.85^**^	–
M	76.07	79.31	83.63	70.22	83.10	15.27	13.84	10.52	10.94	14.22	13.95	11.74	13.30	39.01
SD	10.37	10.66	11.20	16.00	10.52	4.21	4.75	4.88	4.52	4.20	6.34	7.04	5.46	16.71

*n = 234.*

*BFQ E, Big Five Questionnaire Extraversion; BFQ A, Big Five Questionnaire Agreeableness; BFQ C, Big Five Questionnaire Conscientiousness; BFQ ES, Big Five Questionnaire Emotional Stability; BFQ O, Big Five Questionnaire Openness to Experience; DWS SW, Decent Work Scale Safe working conditions; DWS AH, Decent Work Scale Access to healthcare; DWS AC, Decent Work Scale Adequate compensation; DWS FT= Decent Work Scale Free time and rest; DWS CV, Decent Work Scale Complementary Values; OFER AF, The Occupational Fatigue Exhaustion Recovery Acute Fatigue; OFER CF, The Occupational Fatigue Exhaustion Recovery Chronic Fatigue Subscale; OFER-IF, The Occupational Fatigue Exhaustion Recovery Persistent Fatigue Subscale; OFER Tot, The Occupational Fatigue Exhaustion Recovery total score.*

* *p < 0.05;*

** *p < 0.01*.

Table 2 presents the results of the hierarchical linear regressions between the OFER dimensions, the BFQ personality traits, and the DWS. The BFQ personality traits in Step 1 accounted for 13% of the variance in relation to OFER-AF, 16% in relation to OFER-CF, 14% in relation to OFER-IR, and 18% in relation to the OFER total score. In Step 1, the BFQ variables extraversion and emotional stability were found to be statistically significant and negatively associated with all three OFER subscales and the total score and remained statistically significant in Step 2 (confirming H1). In contrast, the BFQ variables of agreeableness, conscientiousness, and openness were not associated with the OFER dimensions in Steps 1 or 2 (confirming H2). In Step 2, the five DWS subscales were entered and explained 20% of the additional variance in relation to OFER-AF, 12% in relation to OFER-CF, 20% in relation to OFER-IR, and 21% in relation to OFER total score. The individual DWS subscales were also found to be statistically significant. The DWS dimension of free time and rest emerged as statistically significant and negatively associated with OFER-AF, OFER-CF, OFER-IR, and OFER total score (confirming H3). Furthermore, the DWS dimension of safe working conditions was found to be statistically significant and negatively associated with OFER-AF, OFER-CF, OFER-IR, and OFER total score (confirming H4). Lastly, negative associations were found between the DWS dimension of adequate compensation and OFER-AF, OFER-CF, OFER-IR, and OFER total score, as well as between the DWS dimension of access to healthcare and OFER-IR.

**Table 2:** Hierarchical regression between Occupational Fatigue Exhaustion Recovery dimensions, Big Five Questionnaire, and Decent Work.

	**OFER acute fatigue**				**OFER chronic fatigue**				**OFER persistent fatigue**				**OFER total score**			
	***R*** ^****2****^ **=** **0.33**				***R*** ^****2****^ **=** **0.28**				***R*** ^****2****^ **=** **0.34**				***R*** ^****2****^ **=** **0.39**			
	β	B	SE B	ΔR^2^	β	B	SE B	ΔR^2^	β	B	SE B	ΔR^2^	β	B	SE B	ΔR^2^
**Step 1**				0.13^***^				0.16^***^				0.14^***^				0.18^***^
BFQ E	−0.19^**^	−0.12	0.04		−0.18^*^	−0.13	0.05		−0.19^**^	−0.10	0.04		−0.21^**^	−0.34	0.11	
BFQ A	0.04	0.02	0.04		−0.07	−0.05	0.05		−0.08	−0.04	0.03		−0.05	−0.07	0.10	
BFQ C	−0.03	−0.02	0.04		−0.13	−0.09	0.04		−0.11	−0.05	0.03		−0.11	−0.16	0.10	
BFQ ES	−0.29^***^	−0.11	0.03		−0.27^***^	−0.12	0.03		−0.25^***^	−0.09	0.02		−0.31^***^	−0.32	0.07	
BFQ O	0.13	0.08	0.04		0.07	0.05	0.05		0.14	0.08	0.04		0.13	0.21	0.11	
**Step 2**				0.20^***^				0.12^***^				0.20^***^				0.21^***^
BFQ E	−0.18^**^	−0.11	0.04		−0.16^*^	−0.12	0.05		−0.19^**^	−0.10	0.03		−0.20^**^	−0.33	0.10	
BFQ A	0.07	0.04	0.04		−0.03	−0.02	0.04		−0.07	−0.04	0.03		−0.01	−0.02	0.09	
BFQ C	−0.02	−0.01	0.04		−0.12	−0.08	0.04		−0.09	−0.04	0.03		−0.09	−0.13	0.09	
BFQ ES	−0.21^***^	−0.08	0.02		−0.21^**^	−0.10	0.03		−0.16^**^	−0.06	0.02		−0.22^***^	−0.23	0.06	
BFQ O	0.07	0.05	0.04		0.04	0.03	0.05		0.09	0.05	0.03		0.08	0.12	0.10	
DWS SW	−0.10	−0.15	0.10		−0.13	−0.22	0.12		−0.14^*^	−0.18	0.08		−0.14	−0.55	0.24	
DWS AH	0.03	0.04	0.08		−0.09	−0.15	0.10		0.03	0.04	0.07		−0.02	−0.06	0.21	
DWS AC	−0.16^**^	−0.20	0.08		−0.15^*^	−0.22	0.09		−0.20^**^	−0.23	0.07		−0.19^**^	−0.64	0.19	
DWS FT	−0.34^***^	−0.47	0.08		−0.17^**^	−0.28	0.10		−0.33^***^	−0.40	0.07		−0.31^***^	−1.15	0.20	
DWS CV	−0.11	−0.17	0.09		−0.03	−0.05	0.12		−0.02	−0.03	0.08		−0.06	−0.25	0.24	

*n = 234.*

*BFQ E, Big Five Questionnaire Extraversion; BFQ A, Big Five Questionnaire Agreeableness; BFQ C, Big Five Questionnaire Conscientiousness; BFQ ES, Big Five Questionnaire Emotional Stability; BFQ O, Big Five Questionnaire Openness to Experience; DWS SW, Decent Work Scale Safe working conditions; DWS AH, Decent Work Scale Access to healthcare; DWS AC, Decent Work Scale Adequate compensation; DWS FT, Decent Work Scale Free time and rest; DWS CV, Decent Work Scale Complementary Values; OFER, Occupational Fatigue Exhaustion RecoveryScale.*

* *p < 0.05;*

** *p < 0.01;*

*** *p < 0.001*.

## Discussion

The purpose of this study was to investigate the specific contribution of decent work characteristics beyond personality traits with respect to occupational fatigue.

The personality traits of extraversion and emotional stability were found to be negative and statistically significantly correlated with all the OFER subscales and the total score. This finding is consistent with the literature (De Vries and Van Heck, 2002; Nater et al., 2010; Kitamura et al., 2013; Poeschla et al., 2013). Emotional stability and extraversion were also statistically significantly associated with lower occupational fatigue in terms of chronic fatigue, acute fatigue, persistent fatigue, and total occupational fatigue (supporting H1). These results confirm the findings of previous literature (De Vries and Van Heck, 2002; Calderwood and Ackerman, 2011; Kitamura et al., 2013; Vassend et al., 2018). In contrast, even though conscientiousness was negatively correlated with OFER-CF, OFER-IR, and OFER total score, this trait was not associated with occupational fatigue in any dimensions. These results are consistent with the findings of Sørengaard et al. (2019). Similarly, openness and agreeableness were not associated with any occupational fatigue dimensions (supporting H2). This finding is in line with prior research that found these personality traits not to be associated with fatigue (Sørengaard et al., 2019) or chronic fatigue (Nater et al., 2010). Furthermore, as expected, the relationships between personality traits and occupational fatigue were consistent for all the dimensions of occupational fatigue (i.e., acute, persistent, chronic, and total) since personality is a stable trait (Costa et al., 2000).

When decent work characteristics were entered into the hierarchical regression models, they were found to be statistically significantly associated with acute, persistent, chronic, and total occupational fatigue (supporting H3). These results are in line with those of Duffy et al. (2021), who observed a direct and negative association between decent work and work-related fatigue.

Sufficient rest and free time were statistically significantly associated with all dimensions of occupational fatigue (supporting H4). This is consistent with Winwood et al. (2005, 2006), who posit that occupational fatigue is strongly associated with employees' ability to have time and rest outside of work. Thus, sufficient rest and free time may allow workers to balance between adaptive fatigue states and recovery from them, maintaining low levels of persistent fatigue that does not progress to a chronic form.

Unexpectedly, adequate compensation was found to be negatively associated with all dimensions of occupational fatigue. This could be explained by the fact that working conditions and the need for survival and power (i.e., essential resources for survival and adaptive levels of control over the needed resources) include adequate compensation (Blustein et al., 2019). Receiving sufficient wages could be related to lower levels of occupational risk, reducing the effects of acute fatigue (Duffy et al., 2019). Further, adequate economic means could satisfy the need for survival and power, enabling more suitable family and social interactions and a higher quality of life, thus facilitating recovery from fatigue during shifts.

Lastly, access to healthcare was found to have a negative association with persistent fatigue only. This finding might be explained by the fact that adequate access to healthcare can sustain employers in maintaining an adequate work–recovery balance (e.g., maintaining sleep hygiene or preventing the primitive response of illness behavior).

## Limitations

The present study has some limitations. First, being a cross-sectional study, it is not possible to analyze changes over time or to establish causality. This is a very key limitation. Future research should investigate which decent work subscales predict occupational fatigue over time by applying longitudinal study designs or ecological momentary assessments. Second, our study enrolled Italian workers. Thus, the results are not generalizable to other countries. Specifically, the Italian context is characterized by low wages and weak wage dynamics that affect many work activities (Di Fabio and Kenny, 2019). This could potentially affect our results, which highlighted adequate compensation as a decent work characteristic associated with occupational fatigue. In addition, regular workers in Italy are protected by rights concerning workplace safety and healthcare (Di Fabio and Kenny, 2019). Furthermore, it is true for our sample that is limited to a single Italian region (i.e., Tuscany) that has an advanced welfare state with a public healthcare system (e.g., Cervia, 2012). Thus, this could also have affected our results, which showed no association between safe working conditions and occupational fatigue. Third, no precarious workers or immigrant workers were enrolled. Future studies are, therefore, necessary to widen the results to these populations, which are characterized by higher levels of marginalization and worse working conditions (Blustein et al., 2018). Fourth, the sample size is small. However, it is sufficient to calculate hierarchical regressions (Tabachnick and Fidell, 2012), and results allow us to provide the first detailed information on the contribution of decent work on personality traits. Fifth, the study enrolled workers from public and private organizations of Tuscany. Future studies could expand our results by comparing different subsamples of workers. For example, considering different types of organizational roles and the years of employment. In fact, severe occupational fatigue and exhaustion were found relatively stable for organizational insiders but slightly dynamic for organizational newcomers and internal job changers (Dunford et al., 2012).

## Conclusions

In conclusion, despite the above-mentioned limitations, our study suggests that decent work is associated with lower occupational fatigue (i.e., acute, chronic, persistent, and total score). Sufficient free time and rest, as well as adequate compensation, showed prominent associations with occupational fatigue. Further, the contribution of the factors embodied in decent work beyond personality traits suggests that decent work could help employees to achieve an optimal work–recovery balance, thereby protecting them from all forms of occupational fatigue. The results could also open future perspectives for interventions in relation to decent work for healthy organizations promoting aspects of organizational justice (not only distributive justice but also procedural justice, interpersonal justice, informational justice) (Colquitt et al., 2001), and policies to guarantee adequate free time and rest for workers to reduce occupational fatigue.

## Data Availability Statement

The raw data supporting the conclusions of this article will be made available by the authors, without undue reservation.

## Ethics Statement

The studies involving human participants were reviewed and approved by Ethics Committee of the Integrated Psychodynamic Psychotherapy Institute (IPPI). The patients/participants provided their written informed consent to participate in this study.

## Author Contributions

ADF conceptualized the paper, supervised and tutored AS. AS and AG reviewed, edited, and wrote all the draft of the paper. AS wrote the first draft of the paper and run statistical analyses. ADF and AG reviewed, edited, and wrote the final draft of the paper. All authors contributed to the article and approved the submitted version.

## Conflict of Interest

The authors declare that the research was conducted in the absence of any commercial or financial relationships that could be construed as a potential conflict of interest.

## Publisher's Note

All claims expressed in this article are solely those of the authors and do not necessarily represent those of their affiliated organizations, or those of the publisher, the editors and the reviewers. Any product that may be evaluated in this article, or claim that may be made by its manufacturer, is not guaranteed or endorsed by the publisher.

## References

[B1] Åkerstedt T. Fredlund P. Gillberg M. Jansson B. (2002). Work load and work hours in relation to disturbed sleep and fatigue in a large representative sample. J. Psychosom. Res. 53, 585–588. 10.1016/S0022-3999(02)00447-6 12127175

[B2] Alarcon G. Eschleman K. J. Bowling N. A. (2009). Relationships between personality variables and burnout: a meta-analysis. Work Stress 23, 244–263. 10.1080/026783709032826008424066

[B3] Autin K. L. Blustein D. L. Ali S. R. Garriott P. O. (2020). Career development impacts of COVID-19: practice and policy recommendations. J. Career Dev. 47, 487–494. 10.1177/0894845320944486

[B4] Bakker A. B. de Vries J. D. (2021). Job Demands–Resources theory and self-regulation: new explanations and remedies for job burnout. Anxiety Stress Copy 34, 1–21. 10.1080/10615806.2020.1797695 32856957

[B5] Bakker A. B. Demerouti E. Dollard M. F. (2008). How job demands affect partners' experience of exhaustion: integrating work-family conflict and crossover theory. J. Appl. Psychol. 93, 901–911. 10.1037/0021-9010.93.4.901 18642992

[B6] Besharat M. A. Behpajooh A. Poursharifi H. Zarani F. (2011). Personality and chronic fatigue syndrome: The role of the five-factor model. Asian J. Psychiatr. 4, 55–59. 10.1016/j.ajp.2010.12.001 23050916

[B7] Blustein D. L. (2006). The Psychology of Working: A New Perspective for Career Development, Counseling, and Public Policy. Mahwah, NJ: Lawrence Erlbaum Associates Publishers.

[B8] Blustein D. L. (2013). The psychology of working: A new perspective for a new era in The Oxford Handbook of the Psychology of working, ed. D. L. Blustein (New York, NY: Oxford University Press), 3–18.

[B9] Blustein D. L. Duffy R. Ferreira J. A. Cohen-Scali V. Cinamon R. G. Allan B. A. (2020). Unemployment in the time of COVID-19: a research agenda. J. Vocat. Behav. 119:103436. 10.1016/j.jvb.2020.103436 32390656 PMC7206417

[B10] Blustein D. L. Kenny M. E. Autin K. Duffy R. (2019). The psychology of working in practice: a theory of change for a new era. Career Dev. Q. 67, 236–254. 10.1002/cdq.12193

[B11] Blustein D. L. Kenny M. E. Di Fabio A. Guichard J. (2018). Expanding the impact of the psychology of working: engaging psychology in the struggle for decent work and human rights. J. Career Assess. 27, 3–28. 10.1177/1069072718774002

[B12] Blustein D. L. Olle C. Connors-Kellgren A. Diamonti A. J. (2016). Decent work: a psychological perspective. Front. Psychol. 7:407. 10.3389/fpsyg.2016.00407 27047430 PMC4806272

[B13] Calderwood C. Ackerman P. L. (2011). The relative impact of trait and temporal determinants of subjective fatigue. Pers. Individ. Differ. 50, 441–445. 10.1016/j.paid.2010.10.030

[B14] Caprara G. V. Barbaranelli C. Borgogni L. Perugini M. (1993). The big five questionnaire: a new questionnaire to assess the five factor model. Pers. Individ. Differ. 15, 281–288. 10.1016/0191-8869(93)90218-R

[B15] Cervia S. (2012). [Democratic paradigm and citizen participation in healthcare: the case of Tuscany]. Paradigma democratico e partecipazione dei cittadini in sanità: il caso della Toscana. Salute e Società 9, 151–164. 10.3280/SES2012-001010

[B16] Choobineh A. Javadpour F. Azmoon H. Keshavarzi S. Daneshmandi H. (2018). The prevalence of fatigue, sleepiness, and sleep disorders among petrochemical employees in Iran. Fatigue: Biomed. Health Behav. 6, 153–162. 10.1080/21641846.2018.1461252

[B17] Colquitt J. A. Conlon D. E. Wesson M. J. Porter C. O. Ng K. Y. (2001). Justice at the millennium: a meta-analytic review of 25 years of organizational justice research. J. Appl. Psychol. 86, 425–445. 10.1037/0021-9010.86.3.425 11419803

[B18] Costa P. T. Jr. Herbst J. H. McCrae R. R. Siegler I. C. (2000). Personality at midlife: Stability, intrinsic maturation, and response to life events. Assessment 7, 365–378. 10.1177/107319110000700405 11151962

[B19] Costa P. T. Jr. McCrae R. R. (1992). Revised NEO Personality Inventory (NEO PI-R and NEO Five-Factor Inventory (NEO FFI): Professional manual. Odessa, FL: Psychological Assessment Resources.

[B20] Costa P. T. Jr. McCrae R. R. (1995). Domains and facets: hierarchical personality assessment using the revised NEO personality inventory. J. Pers. Assess. 64, 21–50. 10.1207/s15327752jpa6401_2 16367732

[B21] Crain T. L. Hammer L. B. Bodner T. Kossek E. E. Moen P. Lilienthal R.. (2014). Work–family conflict, family-supportive supervisor behaviors (FSSB), and sleep outcomes. J. Occup. Health Psychol. 19, 155–167. 10.1037/a0036010 24730425 PMC4145734

[B22] Dansie E. J. Furberg H. Afari N. Buchwald D. Edwards K.. (2012). Conditions comorbid with chronic fatigue in a population-based sample. Psychosomatics 53, 44–50. 10.1016/j.psym.2011.04.001 22221720 PMC3254018

[B23] De Vries J. Van Heck G. L. (2002). Fatigue: relationships with basic personality and temperament dimensions. Pers. Individ. Differ. 33, 1311–1324. 10.1016/S0191-8869(02)00015-6

[B24] Deary V. Chalder T. (2010). Personality and perfectionism in chronic fatigue syndrome: a closer look. Psychol Health. 25, 465–475. 10.1080/08870440802403863 20204923

[B25] Di Fabio A. (2018). Occupational Fatigue Exhaustion Recovery (OFER) Scale: Primo contributo alla validazione della versione italiana [Occupational Fatigue Exhaustion Recovery (OFER) Scale: First contribution to the validation of the Italian version]. Counseling. Giornale Italiano di Ricerca e Applicazioni, 11. Available online at: https://rivistedigitali.erickson.it/counseling/archivio/vol-11-n-1/pokret_im_article-12675/ (accessed June 2, 2021).

[B26] Di Fabio A. Blustein D. L. (2016). Editorial: from meaning of working to meaningful lives: the challenges of expanding decent work. Front. Psychol. 7:1119. 10.3389/978-2-88919-970-9 27512380 PMC4961706

[B27] Di Fabio A. Kenny M. E. (2019). Decent work in Italy: context, conceptualization, and assessment. Pers. Individ. Differ. 110(Part A), 131–143. 10.1016/j.jvb.2018.10.014

[B28] Doerr J. M. Ditzen B. Strahler J. Linnemann A. Ziemek J. Skoluda N.. (2015). Reciprocal relationship between acute stress and acute fatigue in everyday life in a sample of university students. Biol. Psychol. 110, 42–49. 10.1016/j.biopsycho.2015.06.009 26143479

[B29] Duffy R. D. Allan B. A. England J. W. Blustein D. L. Autin K. L. Douglass R. P.. (2017). The development and initial validation of the Decent Work Scale. J. Couns. Psychol. 64, 206–221. 10.1037/cou0000191 28165257

[B30] Duffy R. D. Blustein D. L. Allan B. A. Diemer M. A. Cinamon R. G. (2020). Introduction to the special issue: a cross-cultural exploration of decent work. Pers. Individ. Differ. 116:103351. 10.1016/j.jvb.2019.103351

[B31] Duffy R. D. Blustein D. L. Diemer M. A. Autin K. L. (2016). The psychology of working theory. J. Couns. Psychol. 63, 127–148. 10.1037/cou0000140 26937788

[B32] Duffy R. D. Kim H. J. Gensmer N. P. Raque-Bogdan T. L. Douglass R. P. England J. W.. (2019). Linking decent work with physical and mental health: a psychology of working perspective. Pers. Individ. Differ. 112, 384–395. 10.1016/j.jvb.2019.05.002

[B33] Duffy R. D. Prieto C. G. Kim H. J. Raque-Bogdan T. L. Duffy N. O. (2021). Decent work and physical health: a multi-wave investigation. Pers. Individ. Differ. 127:103544. 10.1016/j.jvb.2021.103544

[B34] Dunford B. B. Shipp A. J. Boss R. W. Angermeier I. Boss A. D. (2012). Is burnout static or dynamic? a career transition perspective of employee burnout trajectories. J. Appl. Psychol. 97, 637–650. 10.1037/a0027060 22309410

[B35] Eurofound (2017). Sixth European Working Conditions Survey—Overview report (2017 update). Luxemburg: Publications Office of the European Union. Available online at: https://www.eurofound.europa.eu/sites/default/files/ef_publication/field_ef_document/ef1634en.pdf

[B36] Glazer S. Gyurak A. (2008). Sources of occupational stress among nurses in five countries. Int. J. Intercult. Relat. 32, 49–66. 10.1016/j.ijintrel.2007.10.003

[B37] Guichard J. (2009). Self-constructing. Pers. Individ. Differ. 75, 251–258. 10.1016/j.jvb.2009.03.004

[B38] Guo F. Wang T. Ning Z. (2017). Subjective measures of work-related fatigue in automobile factory employees. Work 58, 233–240. 10.3233/WOR-172606 28922184

[B39] Hanif Abdul R. Khadizah A.-M. Lin N. (2016). A study into psychosocial factors as predictors of work-related fatigue. Br. J. Nurs. 25, 757–763. 10.12968/bjon.2016.25.13.757 27409786

[B40] International Labour Organization (2015). Decent Work Agenda. Available online at: https://www.ilo.org/global/topics/decent-work/lang--en/index.htm (accessed July 2021).

[B41] Jung M. Lim S. Chi S. (2020). Impact of work environment and occupational stress on safety behavior of individual construction workers. Int. J. Environ. Res. Public Health. 17, 1–21. 10.3390/ijerph17228304 33182704 PMC7696082

[B42] Kachi Y. Inoue A. Eguchi H. Kawakami N. Shimazu A. Tsutsumi A. (2020). Occupational stress and the risk of turnover: a large prospective cohort study of employees in Japan. BMC Public Health. 20:174. 10.1186/s12889-020-8289-5 32019535 PMC7001282

[B43] Kangas M. Montgomery G. H. (2011). The role of cognitive, emotional and personality factors in the experience of fatigue in a university and community sample. Psychol.Health. 1, 1–19. 10.1080/08870440903521779 20945255

[B44] Keinan G. Malach-Pines A. (2007). Stress and burnout among prison personnel: Sources, outcomes, and intervention strategies. Crim. Justice Behav. 34, 380–398. 10.1177/0093854806290007

[B45] Kitamura H. Shindo M. Tachibana A. Honma H. Someya T. (2013). Personality and resilience associated with perceived fatigue of local government employees responding to disasters. J. Occup. Health. 55, 1–5. 10.1539/joh.12-0095-BR 23183020

[B46] Krishnamurthy M. Ramalingam P. Perumal K. Kamalakannan L. P. Chinnadurai J. Shanmugam R.. (2017). Occupational heat stress impacts on health and productivity in a steel industry in Southern India. Saf. Health Work. 8, 99–104. 10.1016/j.shaw.2016.08.005 28344848 PMC5355557

[B47] Kuehn B. (2018). Chronic fatigue care. Jama 320, 750–750. 10.1001/jama.2018.11469 30167707

[B48] Lerman S. E. Eskin E. Flower D. J. George E. C. Gerson B. Hartenbaum N.. (2012). Fatigue risk management in the workplace. J. Occup. Environ. Med. 54, 231–258. 10.1097/JOM.0b013e318247a3b0 22269988

[B49] Liu S. Onwuegbuzie A. J. (2012). Chinese teachers' work stress and their turnover intention. Int. J. Educ. Res. 53, 160–170. 10.1016/j.ijer.2012.03.006

[B50] Lock A. M. Bonetti D. L. Campbell A. D. K. (2018). The psychological and physiological health effects of fatigue. Occup. Med. 68, 502–511. 10.1093/occmed/kqy109 30445654

[B51] Nater U. M. Jones J. F. Lin J. M. S. Maloney E. Reeves W. C. Heim C. (2010). Personality Features and Personality Disorders in Chronic Fatigue Syndrome: A Population-Based Study. Psychother. Psychosom. 79, 312–318. 10.1159/000319312 20664306 PMC2939994

[B52] Nieuwenhuijsen K. Bruinvels D. Frings-Dresen M. (2010). Psychosocial work environment and stress-related disorders, a systematic review. Occup. Med. 60, 277–286. 10.1093/occmed/kqq081 20511268

[B53] Nomaguchi K. M. (2009). Change in work-family conflict among employed parents between 1977 and 1997. J. Marriage Fam. 71, 15–32. 10.1111/j.1741-3737.2008.00577.x

[B54] Peiró J. M. Tetrick L. (2011). Occupational health psychology in IAAP Handbook of Applied Psychology, eds. P. R. Martin, F. M. Cheung, M. C. Knowles, M. Kyrios, L. Littlefield, J. B. Overmier et al. (Chichester: Wiley Blackwell). 292–315.

[B55] Poeschla B. Strachan E. Dansie E. Buchwald D. S. Afari N. (2013). Chronic fatigue and personality: a twin study of causal pathways and shared liabilities. Ann. Behav. Med. 45, 289–298. 10.1007/s12160-012-9463-5 23361410 PMC3643988

[B56] Preckel D. Känel R. V. Kudielka B. M. Fischer J. E. (2005). Overcommitment to work is associated with vital exhaustion. Int. Arch. Occup. Environ. Health. 78, 117–122. 10.1007/s00420-004-0572-8 15726394

[B57] Reichl C. Leiter M. P. Spinath F. M. (2014). Work–nonwork conflict and burnout: a meta- analysis. Hum. Relat. 67, 979–1005. 10.1177/0018726713509857

[B58] Reynolds K. J. Vernon S. D. Bouchery E. Reeves W. C. (2004). The economic impact of chronic fatigue syndrome. Cost Eff. Resour. Alloc. 2:4 10.1186/1478-7547-2-4 15210053 PMC449736

[B59] Ricci J. A. Chee E. Lorandeau A. L. Berger J. (2007). Fatigue in the U.S. workforce: prevalence and implications for lost productive work time. J. Occup. Environ. Med. 49, 1–10. 10.1097/01.jom.0000249782.60321.2a 17215708

[B60] Richter K. Acker J. Adam S. Niklewski G. (2016). Prevention of fatigue and insomnia in shift workers—a review of non-pharmacological measures. EPMA J. 7:16. 10.1186/s13167-016-0064-4 27486484 PMC4970219

[B61] Rose D. M. Seidler A. Nübling M. Latza U. Brähler E. Klein E. M.. (2017). Associations of fatigue to work-related stress, mental and physical health in an employed community sample. BMC Psychiatry 17:167. 10.1186/s12888-017-1237-y 28476149 PMC5420158

[B62] Rosekind M. R. Gregory K. B. Mallis M. M. Brandt S. L. Seal B. Lerner D. (2010). The cost of poor sleep: workplace productivity loss and associated costs. J. Occup. Environ. Med. 52, 91–98. 10.1097/JOM.0b013e3181c78c30 20042880

[B63] Roy-Byrne P. Afari N. Ashton S. Fischer M. Goldberg J. Buchwald D. (2002). Chronic fatigue and anxiety/depression: a twin study. Br. J. Psychiatry. 180, 29–34. 10.1192/bjp.180.1.29 11772848

[B64] Rutledge D.N. Douville S. Winokur E. Drake D. Niedziela D. (2021). Impact of engagement factors on nurses' intention to leave hospital employment. J. Nurs. Manag. 10.1111/jonm.13287 33606341

[B65] Sabir A. A. Isha A. S. N. B. (2016). Assessing the fatigue related psychological risk factors among oil and gas tankers drivers in Malaysia. Int. Rev. Manag. Mark. 6, 138–142. Available online at: http://www.econjournals.com/index.php/irmm/article/view/2478 (accessed August 14, 2021).

[B66] Sagherian K. Geiger-Brown J. Rogers V. E. Ludeman E. (2019). Fatigue and risk of sickness absence in the working population: a systematic review and meta-analysis of longitudinal studies. Scand. J. Work Environ. Health. 45, 333–345. 10.5271/sjweh.3819 30937459

[B67] Saksvik-Lehouillier I. Bjorvatn B. Hetland H. Sandal G. M. Moen B. E. Magerøy E.. (2012). Personality factors predicting changes in shift work olerance: a longitudinal study among nurses working rotating shifts. Work Stress. 26, 143–160. 10.1080/02678373.2012.686344

[B68] Somantri I. Yuliati M. Winwood P. Adiningsih A. (2020). Work-related fatigue among impatient unit nurses. J. Nurs. Care. 3, 199–295. 10.24198/jnc.v3i3.22286

[B69] Sørengaard T. A. Saksvik-Lehouillier I. Langvik E. (2019). Longitudinal and cross-sectional examination of the relationship between personality and fatigue among shift workers. Cogent Psychol. 6, 1574095. 10.1080/23311908.2019.1574095

[B70] Tabachnick B. G. Fidell L. S. (2012). Using Multivariate Statistics, 6th Edn. Boston, MA: Pearson.

[B71] Techera U. Hallowell M. Stambaugh N. Littlejohn R. (2016). Causes and consequences of occupational fatigue: meta-analysis and systems model. J. Occup. Environ. Med. 58, 961–973. 10.1097/jom.0000000000000837 27525527

[B72] Thorsteinsson E. B. Brown R. F. (2009). Mediators and moderators of the stressor–fatigue relationship in nonclinical samples. J. Psychosom. Res. 66, 21–29. 10.1016/j.jpsychores.2008.06.010 19073289

[B73] Tucker P. (2003). The impact of rest breaks upon accident risk, fatigue and performance: a review. Work Stress. 17, 123–137. 10.1080/0267837031000155949

[B74] Valero S. Sáez-Francàs N. Calvo N. Alegre J. Casas M. (2013). The role of neuroticism, perfectionism and depression in chronic fatigue syndrome: a structural equation modeling approach. Compr. Psychiatry. 54, 1061–1067. 10.1016/j.comppsych.2013.04.015 23759150

[B75] Vassend O. Røysamb E. Nielsen C. S. Czajkowski N. O. (2018). Fatigue symptoms in relation to neuroticism, anxiety-depression, and musculoskeletal pain. A longitudinal twin study. PLoS One 13. 10.1371/journal.pone.0198594 PMC5991664 29879175

[B76] Williamson A. Friswell R. (2013). Fatigue in the workplace: causes and countermeasures. Biomed. Health Behav. 1, 81–98. 10.1080/21641846.2012.744581

[B77] Winwood P. C. Lushington K. Winefield A. H. (2006). further development and validation of the occupational fatigue exhaustion recovery (OFER) scale. J. Occup. Environ. Med. 48, 381–389. 10.1097/01.jom.0000194164.14081.06 16607192

[B78] Winwood P. C. Winefield A. H. Dawson D. Lushington K. (2005). Development and validation of a scale to measure work-related fatigue and recovery: the occupational fatigue exhaustion/recovery scale (OFER). J. Occup. Environ. Med. 47, 594–606. 10.1097/01.jom.0000161740.71049.c4 15951720

[B79] Yun Y. H. Lee M. K. Chun H. N. Lee Y. M. Park S. M. Mendoza T. R.. (2008). Fatigue in the general korean population: application and normative data of the brief fatigue inventory. J. Pain Symptom Manag. 36, 259–267. 10.1016/j.jpainsymman.2007.10.016 18411013

[B80] Zbryrad T. (2009). Stress and professional burn-out In selected groups of workers. Informatologia 42, 186–191.

[B81] Zhang F. Wu C. Jia C. Gao K. Wang J. Zhao H.. (2019). Artificial intelligence based discovery of the association between depression and chronic fatigue syndrome. J. Affect. Disord. 250, 380–390. 10.1016/j.jad.2019.03.011 30877861

